# Medical students’ attitudes towards older persons – a systematic review and meta-analysis

**DOI:** 10.1080/10872981.2026.2661455

**Published:** 2026-04-21

**Authors:** Ali Vahedi, Åsa G. Andersson, Brynjar Fure

**Affiliations:** a Department of Internal Medicine, Section of Geriatric Medicine, Central Hospital Karlstad, Region Värmland, Sweden; b School of Medical Sciences, Faculty of Medicine and Health, Örebro University, Örebro, Sweden; c Department of Geriatrics, School of Medical Sciences, Faculty of Medicine and Health, Örebro University, Örebro, Sweden

**Keywords:** Medical students, attitudes, older persons, systematic review, medical education

## Abstract

**Objective:**

Ageism negatively affects older people’s health, well-being, and quality of care. Identifying ageism and attitudes toward older adults may help identify medical students motivated to work with a growing older population. We conducted a systematic review of interventions to reduce ageism among medical students.

**Methods:**

We conducted a systematic literature retrieval in Medline, the Cochrane Library, Epistemonikos and PubMed via Ovid, following the Preferred Reporting Items for Systematic Reviews and Meta-Analyses, from database inception to August 23rd, 2024. We searched for studies examining medical students’ attitudes towards older persons, including interventions to influence these attitudes.

**Results:**

The systematic review included 21 studies. Study samples ranged between 29 and 415, with a pooled total of 3940 medical students, the majority conducted in the USA. The meta-analysis of students’ attitudes before and after all interventions included a total of 2004 students, yielding a non-significant standardized mean difference (SMD) of 0, 12 (95% CI −0, 01 to 0, 26). The meta-analysis of students’ attitudes before and after all interventions (35–37, 39, 41, 45, 48, 50), after removing UCLA-GAS and R-GAS, included a total of 1344 students with a standardized mean difference of 0, 23 (95% CI 0, 06 to 0, 40). Analysis of empathy-based teaching interventions included 1652 students, and showed a statistically significant effect, SMD 0, 18 (95% CI 0, 01 to 0, 34). Analysis of 352 students receiving traditional teaching methods yielded no effect on ageism, with an SMD −0, 13 (95% CI −0, 81 to 0, 54). High heterogeneity (I^2^ 75—85%) affects our confidence in the effect estimates.

**Conclusion:**

Empathy-based interventions seem to improve medical students’ attitudes towards older persons. Traditional teaching methods may increase negative attitudes. Considering the high heterogeneity, the results should be interpreted cautiously. Adding empathy-based components to medical curricula could combat ageism. More studies are needed to examine whether the results of studies from North America are valid globally.

## Introduction

### The older person

There is no universal definition of who an older person is [1]. Worldwide, definitions are often based on chronological age, such as the United Nations’s definition that an older person is >60 years of age [2] or guidelines for clinical studies that define an older person as >65 years [3]. In a previous study, older persons perceived old age as beginning at the age of 74, and their perception was affected by a decline in health, physical limitations, decreased independence, social convention, and a change in physical appearance [4]. The older the persons were, the later they perceived the beginning of old age.

In healthcare, older people are often viewed as the geriatric population [5]. While geriatric syndromes may present from 65 years of age or even earlier in life, they are more prevalent from 80 years onwards [5]. Still, older persons are, as a group, inhomogeneous [6]. Individuals may be robust or frail status, present with multiple diagnoses or none, and with varying degrees of independence in activities of daily functions [6].

### Ageism

Ageism describes the discrimination of persons of any age [7], though the term is often used to describe the discrimination of older persons [8]. Ageism may be intentional or unintentional, may occur between persons or structural [9], and may impact older persons negatively by potentially causing impaired health, well–being, and even quality of health care received [8]. Being an older person, having a lower healthy life expectancy and working in specific trades, such as in the hospitality sector, increases the risk of being a target of ageism [9]. Among young people, ageism may be associated with aging anxiety, fear of death, lack of communication with older persons and a lack of knowledge [10]. The factors that seem to increase the risk of negative attitudes against older persons are young age, male sex and lower education levels, while positive personality traits and intergenerational contact decrease the risk [9]. Ageism is frequently related to other forms of stereotypes, prejudice and discrimination, further increasing the negative impact of discrimination [9].

### Medical students and ageism

While interventions have shown that ageism among medical students can be reduced by increased exposure to the geriatric discipline in medical school, internationally, the time devoted to geriatric studies is brief [11]. Previous studies that evaluated mean hours assigned for geriatric teaching and training in medical schools reported that medical students in Canada spent a mean of 82 h, in Sweden 59 h, in the United Kingdom 55, 5 h, and in the United States of America 14, 4 h [11,12].

A previous systematic review showed that medical students were less inclined to present ageism if the quality of their previous relationships with older persons had been positive [13]. The authors also presented a link between intrinsic motivation and attitudes towards older persons, and identifying ageism may help screen for medical students who are motivated to work with older patients in healthcare [13]. Recent studies have indicated that medical students presenting ageism provide negative evaluations not only toward older persons, but also toward other groups, and that education in ageism alone may not be enough, as the students’ personalities should also be considered as well [10,13]. However, previous systematic reviews were not performed recently, and while they do analyze statistical significance, they do not include effect estimates.

Ageism causes medical professionals to overlook the severity of ailments, thus delaying or preventing adequate interventions [14]. This leads to unequal treatment, increases the risk of inappropriate diagnoses, inadequate management of health conditions and substandard health‐care decisions. Additionally, ageism hinders effective communication between health‐care professionals and older patients, causing misunderstandings, poorer patient satisfaction, and a lack of adherence to treatment plans, and prevents older adults from seeking necessary care [14].

### Instruments used to assess attitudes towards older persons

The aging semantic differential (ASD) by Rosencrantz and McNevin is among the most widely used attitude measuring instruments in geriatric and gerontological research [15]. The ASD is a 32-item questionnaire assessing the stereotypic attitudes of young people towards older adults. The respondent agrees or disagrees with each statement on a 6-point Likert scale. The ASD measures three major dimensions: instrumental-ineffective, autonomous-dependent, and personal acceptability–unacceptability [16]. Higher scores indicate more negative attitudes towards older persons.

The University of California, Los Angeles Geriatrics Assessment Scale (UCLA–GAS) is another widely used instrument [17]. The UCLA–GAS is a 14-item survey with responses indicating agreement or disagreement with a statement [18]. The instrument uses a Likert–scale format with five positively and nine negatively worded statements rated on a scale from 1 (strongly disagree) to 5 (strongly agree), with 3 representing a neutral rating. The scores of the negatively worded items are reversed when the total score is calculated, and a higher score indicates a positive attitude towards older persons.

Kogan’s Attitudes Towards Old People Scale (OP) [19] is a frequently used attitude instrument in research [20]. It consists of 34 items assessing positive and negative attitudes toward older persons. Lower scores indicate more favorable attitudes.

The Carolina Opinions on Care of Older Adults (COCOA) scale [21] is a 42-item questionnaire in which respondents agree or disagree with a statement on a 5-point scale. The instrument assesses early interests in and attitudes towards geriatrics, empathy and compassion, ageism, clinical and social services for, and the social value of older adults. Higher scores reflect more positive attitudes toward older persons.

The Fraboni Scale of Ageism (FSA) [22] comprises 29 items representing beliefs regarding the antilocution, avoidance and discrimination of the elderly. The respondent agrees or disagrees with each statement on a 6-point scale, and some questions are scored reversely. Higher scores indicate negative attitudes towards older persons.

The Maxwell Sullivan Attitude Scale (MSAS) [23] is a 28-item survey with statements regarding attitudes and educational preparedness to manage older patients. The statements are answered on a 5-point scale, and lower scores indicate positive attitudes.

Alford et al. [24] used a locally developed survey. In addition to questions about attitudes, the instrument also assesses careers in geriatrics and clinical practice. Alford et al. (24) use part I of this instrument, including 28 statements about older people as people, as patients and about geriatrics in general. The respondent agrees or disagrees with the statement on a 6-point scale, and a higher score indicates a more positive attitude towards older persons.

The 25-item Geriatrics Attitude Survey was developed by Warren et al. [25], and was based on Kafer’s Aging Opinion Survey [26] and the Maxwell Sullivan Attitude Scale. The statements are positively or negatively loaded and are answered on a 4-point Likert scale, with higher scores indicating a positive attitude.

It is widely regarded that the scales used in the review measure ageism by exploring the negative attitudes, beliefs and stereotypes of older persons [27].

### Interventions

A previous systematic review examined traditional teaching methods and empathy-based interventions [28]. Interventions that encourage participants to relate to or share experiences with older persons outside the medical setting may increase empathy. Interventions including an empathy-building component may be more likely to improve attitudes compared to traditional teaching [28]. Mentorship programs between community–dwelling healthy older people and medical students [13] and experimental programs including the psychology of aging and sensory loss-activities have been shown to be promising [25,29], as well as computer software for exploring patient-oriented goals and preferences, the appropriateness or futility of medical care, and the costs associated with medical care [30].

### Aim

The ongoing demographic shift, with the proportion of older persons in society increasing globally [31], leads to a need for more physicians willing to work with older adults and more specializing in geriatric medicine. We aimed to perform a systematic review and meta-analysis to assess interventions designed to reduce ageism among medical students.

## Methods

### Inclusion and exclusion criteria

This systematic review was conducted and reported in accordance with the Preferred Reporting Items for Systematic Reviews and Meta-Analyses (PRISMA) (16).

PICOS.

P: Medical students.

I: Interventions designed to influence medical students’ negative attitudes towards older people.

C: Medical students are not exposed to interventions designed to affect their attitudes towards older people.

O: Prevalence of and factors associated with negative and positive attitudes towards older people. Change in negative attitudes towards older people.

S: Observational studies with or without a control group. Randomized controlled studies.

Exclusion criteria: Studies that do not examine medical students, include measures of statistical dispersion, include quantitative measures of attitudes or examine medical students’ attitudes, and where medical students are not stratified into a separate group, and for which the presented results are not suitable for meta-analysis. Studies not written in English were excluded.

### Search strategy

A trained information specialist conducted a systematic literature retrieval in Medline, the Cochrane Library, Epistemonikos, and PubMed through Ovid from the date of inception of the database until August 23rd, 2024. We manually sorted references, citations, and other related publications from the articles after the first retrieval and searched the reference list of systematic reviews for relevant studies that included results suitable for meta-analysis and where the results of medical student attitudes were stratified into a separate group. We used MeSH terms and free terms as search terms, combined with a Boolean conjunction. For the complete search strategy, see Supplementary material 1.

Full-text articles were eligible if they were published in a peer-reviewed international journal written in English. If several studies used the same cohort, we opted to limit inclusion to the one with the largest sample size. Studies with qualitative questionnaires, systematic reviews, abstracts and editorial letters were excluded.

The present systematic review and meta‐analysis were registered in the International Prospective Register of Systematic Reviews, PROSPERO (CRD42023481026).

### Study selection and data extraction

Data were collected regarding age and sex, year of publication, country of the study, setting, study design, number of included medical students, year in medical school, and outcomes of questionnaires exploring the attitudes towards older persons. Data were extracted through the standardized PRISMA checklist, including title, abstract, introduction, methods, results, discussion, and funding.

We classified interventions as knowledge-building or empathy-building. Knowledge–building interventions were defined as consisting of lectures on geriatrics or clinical competence and usually had a medical focus. Empathy-building interventions stimulate relationships and experience-sharing with older adults outside of a medical setting, for example, with aging simulation exercises and contact with healthy older adults.

All authors reviewed the systematic literature retrieval, screened references, and assessed abstracts and full-text articles. AV manually searched the reference lists of systematic reviews for additional articles. In cases of disagreement, inclusion was decided through consensus. AV, ÅA, and BF independently extracted data in accordance with the PRISMA checklist. AV, ÅA, and BF performed the quality assessment of each study using the checklist and overall quality assessment across studies of the pooled estimates. Disagreements were solved through consensus.

### Quality assessment

Quality assessment of the included studies was performed using the Critical Appraisal Skills Programme (CASP) checklist for cross–sectional studies [32] and the GRADEpro software for evaluating the certainty of the effect estimates across included studies [33].

### Statistical analysis

We extracted reported data, specifically mean attitude scores with Standard Deviation (SD)standard deviation (SD), across all studies. The extracted data were entered into Review Manager 5.4, and we performed a meta-analysis of the mean difference or standardized mean difference with confidence intervals. The mean difference was used in the meta-analyses when the same attitude instrument had been applied in the included studies; otherwise, the standardized mean difference was used. A sensitivity analysis was performed in the pooled analysis, excluding instruments believed to measure beliefs rather than attitudes. We applied random effects and performed I² as a measure of heterogeneity.

## Results

### Characteristics of included studies

**Figure 1.**
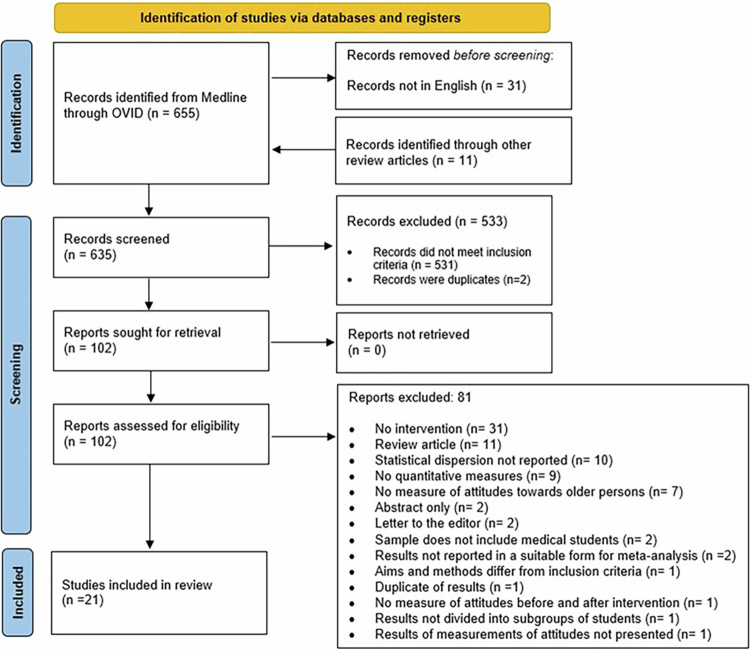
Prisma flow diagram of the search process.

A total of 655 records were identified in the literature search, and an additional 11 records were identified through reading reference lists of previous systematic reviews. After the first screening, 102 articles were sought for retrieval, and after full-text reading, 21 studies were included in the systematic review (see Figure 1). For a complete list of excluded studies, see Supplementary material 2.

Of the 21 studies included [24, 25, 29, 30, 34−50], all were performed in medical faculties at universities in Western countries, with the majority (76, 2%) in the USA. The publishing years ranged between 1983 and 2024. The curriculum consisted of 4-year programs preceded by 2 years of pre-clinical courses. The most common screening tools used for measuring attitudes towards older persons were the ASD (38, 1%), followed by the UCLA GAS (33, 3%) and the OP (14, 3%). Study samples ranged between 29 and 415, with a pooled total of 3940 medical students. Interventions were specifically designed to positively impact ageism, such as through various senior programs, settings designed to improve contacts with older persons, as well as through computer software, and as part of the medical program curriculum in the form of standard or custom geriatrics courses (see Table 1). Among studies performing the intervention year 1 or 2, 66, 7% saw a positive effect on students’ attitudes, compared to 71, 4% for interventions year 3 or 4, and 60% for longitudinal interventions.

**Table 1.** Characteristics of included studies.

Author^a^	Yeara	Title	Country	Study design	Sample, N	Mean age (SD)^b^, if not otherwise specified	Gender, F (%)	Education, yrs	Intervention	Attitude scale
Adelman, RD et al.	1992	Geriatric education Part II: The effect of a Well Elderly Program on Medical Student Attitudes toward Geriatric Patients	USA	Cross-sectional	93	NR	39, 8%	Year 4	Well Elderly Program	ASD
Alford, CL et al.	2001	An Introduction to Geriatrics for First-Year Medical Students	USA	Cross-sectional	203	NR	52, 4%	Year 1	Geriatrics Continuity of Care Track	Locally developed survey
Biasio, JC et al.	2016	Longitudinal assessment of medical student attitudes toward older people	USA	Longitudinal intervention	404	23, 8 (2, 5)	44, 6%	Years 1–4	Geriatrics clinical experience and clerkship	UCLA GAS
Duke, *P* et al.	2009	Using a geriatric mentoring narrative program to improve medical student attitudes towards the elderly	USA	Cross-sectional	55	20–30 years	49, 1%	Year 1	Geriatric narrative mentoring program	R–GAS
Edwards, MJ et al.	1996	Attitudes to and knowledge about elderly people: a comparative analysis of students of Medicine, English and Computer Science and their teachers	UK	Cross-sectional	362	NR	NR	Year 4	Two–week geriatric module	ASD
Eskildsen, MA et al.	2009	A Multimodal Aging and Dying Course for First-Year Medical Students Improves Knowledge and Attitudes	USA	Cross-sectional	130	NR	49, 2%	Year 1	Week–long module on aging	UCLA GAS
Gonzales, E et al.	2010	Changing Medical Students' Attitudes Toward Older Adults	USA	Cross-sequential study	328	24 (2, 4)	67% treatment group, 55% control	Years 1 and 4	Vital Visionaries intergenerational program	Refined–ASD
Hughes, NJ et al.	2008	Medical Student Attitudes Toward Older People and Willingness to Consider a Career in Geriatric Medicine	UK	Cross-sequential study	169	Year 1: < 20 years 77, 6%	55, 2%	Years 1 and 4	Intensive geriatric medicine program	UCLA GAS
Intrieri, RC et al.	1993	Improving Medical Students’ Attitudes Toward and Skills with the Elderly	USA	Cross-sectional	96	NR	25%	Year 3	Gerontological experimental program	ASD
Jeste, DV et al.	2018	Effect of Short–Term Research Training Programs on Medical Students’ Attitudes Toward Aging	USA	Cross-sectional	134	NR	55, 7%	Year 1	MSTAR and M–STREAM programs	COCOA
Kusumastuti, S et al.	2017	When Contact Is Not Enough: Affecting First Year Medical Students' Image towards Older Persons	Netherlands	Cross-sectional	415	18 years (42, 7%)	65.3%	Year 1	Two–week care internship	ASD, AOP
Lu, W–H et al.	2010	First Year Medical Students' Knowledge, Attitudes, and Interest in Geriatric Medicine	USA	Cross-sectional	192	23 (1.97)	54.7%	Year 1	The Senior Teacher Education Partnership (STEP)	ASD
Mendoza de la Garza, M et al.	2018	Evaluation of the Impact of a senior mentor program on medical students’ geriatric knowledge and attitudes toward older adults	USA	Cross-sequential	171	NR	NR	Year 1 and 2	Senior Sage program	UCLA GAS
Morgan, S et al.	2024	Combating ageism in medical education with narrative medicine	USA	Cross-sectional	151	NR	NR	Year 4	My Life, My story interview	UCLA GAS
Powell, FC et al.	1988	Stability of Medical Students’ Attitudes Toward Aging and Death	USA	Longitudinal intervention	277	23, 7 years	21%	Year 1 and 4	Medical school experience	OP
Ruiz, JG et al.	2015	Group-Based Differences in Anti-Aging Bias Among Medical Students	USA	Longitudinal intervention	103	24, 81 (2, 31)	50, 0%	Year 1-4	IAT (?)	FSA
Sainsbury, R et al.	1992	Attitudes of medical students to old people: a cross–sectional national comparative study	New Zealand	Cross-sectional	150	NR	NR	Year 4	Five–week clinical attachment	ASD
Shue, CK et al.	2005	Changing Medical Students’ Attitudes about Older Adults and Future Older Patients	USA	Longitudinal intervention	161	NR	NR	Year 1	Mentors–on–aging program	ASD, MSAS
van de Pol, MH et al.	2014	Teaching Geriatrics Using an Innovative, Individual–Centered Educational Game: Students and Educators Win. A Proof–of- Concept Study	Netherlands	Cross-sectional	29	23, 0(1, 5) intervention, 24, 0 (1, 0) control	51, 7% intervention, 33, 3% control	Year 3	GeriatriX game software	ASD
Warren, DL et al.	1983	Effects of Geriatric Education on the Attitudes of Medical Students	USA	Cross-sectional	80	28 (?)	21%	Year 3	25-hour Geriatrics educational program	Geriatrics Attitude Survey
Westmoreland, GR et al.	2009	Improving Medical Student Attitudes Toward Older Patients Through a ‘Council of Elders’ and Reflective Writing Experience	USA	Cross-sectional	237	NR	NR	Year 1 or 2	Council of Elders and Reflective Writing program	UCLA GAS

Key: AOP = Attitudes toward Old People, ASD = Aging Semantic Differential, COCOA = Carolina Opinions on Care of Older Adults, FSA = Fraboni Scale of Ageism, HPBOE = Health Professional Beliefs and Opinions about Elders, MSAS = Maxwell Sullivan Attitude Scale, MSTAR = Medical Student Training in Aging Research Program, MSTREAM = Medical Students’ Sustained Training and Research Experience in Aging and Mental Health, NR = Not reported, OP = Kogan’s Attitudes Towards Old People Scale, R–GAS = University of California Los Angeles Geriatrics Attitudes Scale for primary care residents, SDS = Semantic Differential scale, UCLA GAS = University of California Geriatrics Assessment Scale

Note: All studies were performed at medical programs at universities.

^a^ 1 = Corresponding author,

^b^ 2 = control group.

### Meta-analysis

We grouped all 21 studies and intervention methods into one analysis (17–37), followed by separate analyses of 17 studies evaluating empathy-based teaching interventions and four studies evaluating traditional teaching methods, all using standardized mean difference. In addition, separate analyses using the mean difference were performed for the studies that applied the two most common attitude scales, namely, the Ageing Semantic Differential (ASD), six studies, and the University of California Los Angeles Geriatrics Attitudes Scale (UCLA–GAS), six studies.

We analyzed the results of the ASD questionnaire from Adelman et al. [34], Edwards et al. [38], Intrieri et al. [29], Lu et al. [44], Sainsbury et al. [48], van de Pol et al. [30] and Kusumastuti et al. [43] and the refined-ASD from Gonzales et al. [40]. For de Biasio et al. [35], de la Garza et al. [36], Eskildsen et al. [39], Hughes et al. [41], Morgan et al. [45], Westmoreland et al. [50] and Sainsbury et al. [48], we used the UCLA–GAS, and for Duke et al. [37], we used the R–GAS. Alford et al. [24] used a locally developed survey, Jeste et al. [42] used the Carolina Opinions on Care of Older Adults (COCOA) scale, Powell et al. [46] the Kogan’s Attitudes Towards Old People Scale (OP), Ruiz et al. [47] used the Fraboni Scale of Ageism (FSA), Warren et al. [25] used the Geriatrics Attitude Survey and Shue et al. [49] the Maxwell Sullivan Attitude Scale (MSAS).

**Figure 2.**
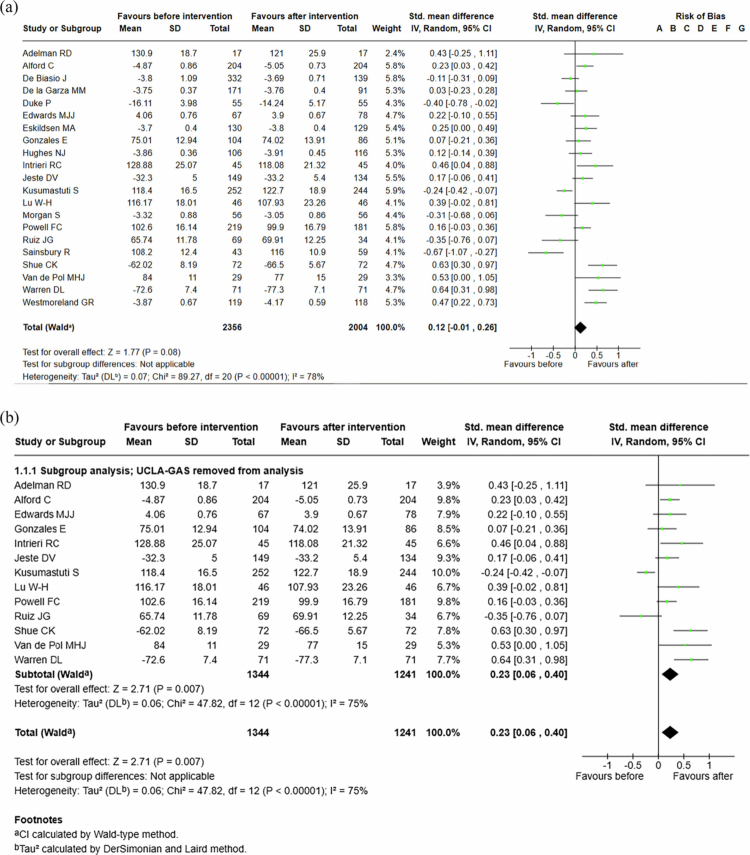
(a) Students’ attitudes before and after the interventions, all studies, both traditional and empathy-based teaching interventions. b. Students’ attitudes before and after interventions, UCLA-GAS and R-GAS were removed from analysis, both traditional and empathy-based teaching interventions.

The meta-analysis of students’ attitudes before and after all interventions [24, 25, 29, 30, 34−50] included a total of 2004 students and showed a standardized mean difference of 0, 12 (95% CI −0, 01 to 0, 26). Across all interventions, student attitudes improved, though the results were not statistically significant (see Figure 2a).

The meta-analysis of students’ attitudes before and after all interventions [35−37, 39, 41, 45, 48, 50], after removing UCLA-GAS and R-GAS, included a total of 1344 students and showed a standardized mean difference of 0, 23 (95% CI 0, 06 to 0, 40). Across all interventions, student attitudes were significantly improved (see Figure 2b).

**Figure 3.**
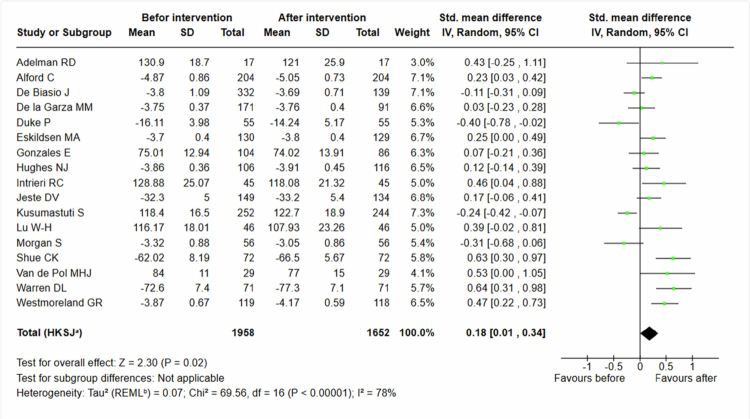
Students’ attitudes, empathy-based teaching interventions only.

The analysis of empathy-based teaching interventions included 1652 students after the intervention [24, 25, 29, 30, 34−37, 39−45, 49, 50] and showed a statistically significant effect with a standardized mean difference of 0, 18 (95% CI 0, 01 to 0, 34) (see Figure 3).

The interventions included customized geriatric medicine programs [35,41], a module on aging [39], and a care internship [43]. Two medical schools performed experimental programs, including the psychology of aging and sensory loss–activities, as well as interacting with older adults [25,29], one used the GeriatriX game software [30] and the remainder of various programs based on contact with older community-dwelling persons [36,37,40,42,44,45,49,50].

For a comprehensive list of interventions, see Table 1.

**Figure 4.**
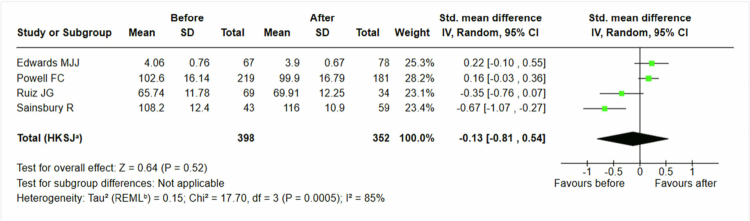
Students’ attitudes. Traditional teaching interventions only.

We analyzed 352 students receiving traditional teaching methods [38, 46−48] and found no effect on ageism, standardized mean difference −0, 13 (95% CI −0, 81 to 0, 54), see Figure 4. In contrast, traditional teaching methods seemed to increase negative attitudes toward older people.

**Figure 5.**
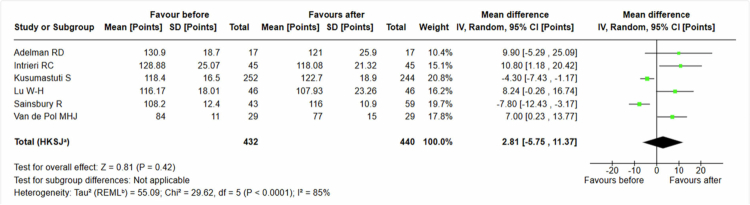
Students' attitudes. Ageing Semantic Differential (ASD) only.

We introduced data from six studies evaluating empathy-based interventions [29,30,34,43,44,48], with the exception of Sainsbury et al. [48], of 440 students assessed with ASD. We observed a non-significant positive increase in the effect on the students’ attitudes, with a mean difference of 2, 81 (95% CI −5, 75 to 11, 37), see Figure 5.

**Figure 6.**
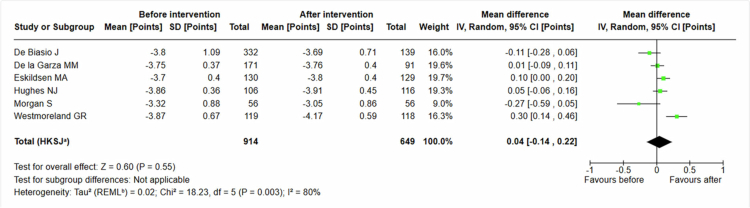
Students' attitudes. UCLA-GAS.

We introduced data from six studies [35,36,39,41,45,50] evaluating empathy-based interventions of 649 students assessed with the UCLA-GAS (see Figure 6). We found no statistically significant effect; the mean difference 0, 04 (95% CI −0, 14 to 0, 22).

**Figure 7.**
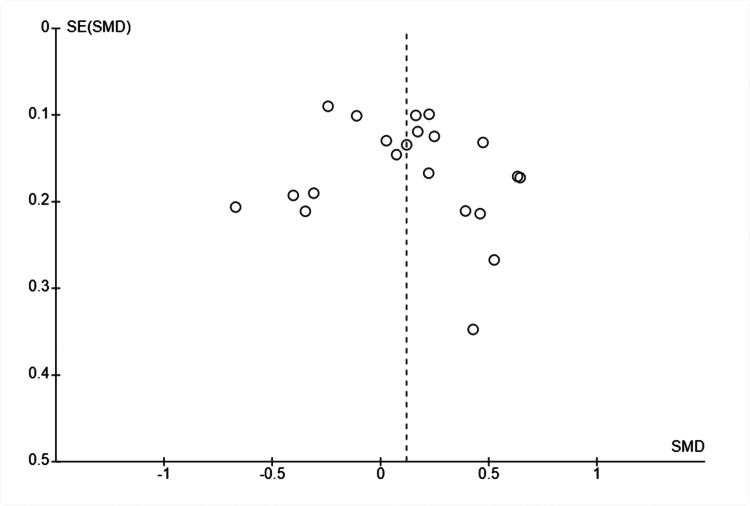
Funnel plot. All studies.

We observed no significant signs of publication bias or systematic differences in precision (see Figure 7). Although the funnel plot was not perfectly symmetrical, most studies were located within a pyramid corresponding to where 95% are expected to lie in the absence of publication bias.

### Quality assessment

Using the CASP checklist, the studies were appraised to have a low risk of bias (see Supplementary material 3). Power calculations were not reported in most studies, but the sample sizes were deemed adequate for the research question and study type.

### Certainty of the results

GRADE assessment of overall effect estimates showed low confidence in the effect estimates, due to non-randomized studies, inconsistency with results pointing in different directions, and imprecision related to wide confidence intervals; see Table 2.

**Table 2.** Certainty of the estimates across all included studies according to the grading of recommendations assessment, development and evaluation (GRADE).

Certainty assessment							No of patients		Effect			
№ of studies	Study design	Risk of bias	Inconsistency	Indirectness	Imprecision	Other considerations	Before	After	Relative (95% CI)	Absolute (95% CI)	Certainty	Importance
**Both. empathy-based, geriatric teaching interventions and traditional geriatric teaching**												
21	non-randomized studies	not serious	serious^a^	not serious	serious^b^	none	2356	2004	SMD **0.12 SD higher** (0.01 lower to 0.26 higher)	⨁◯◯◯ Very low^a,b^	IMPORTANT	
**Empathy-building, geriatric teaching interventions only**												
17	non-randomized studies	not serious	serious^a^	not serious	not serious	none	1958	1652	–	SMD **0.18 SD higher** (0.01 higher to 0.34 higher)	⨁◯◯◯ Very low^a^	IMPORTANT
**UCLA-GAS**												
6	non-randomized studies	not serious	serious^a^	not serious	serious^b^	none	914	649	–	MD **0.04 points higher** (0.14 lower to 0.22 higher)	⨁◯◯◯ Very low^a,b^	IMPORTANT
**ASD**												
6	non-randomized studies	not serious	not serious	not serious	serious^b^	none	432	440	–	MD **2.81 points higher** (5.75 lower to 11.37 higher)	⨁◯◯◯ Very low^b^	IMPORTANT
**Traditional geriatric teaching only**												
4	non-randomized studies	not serious	serious^a^	not serious	serious^b^	none	398	352	–	SMD **0.13 SD lower** (0.81 lower to 0.54 higher)	⨁◯◯◯ Very low^a,b^	IMPORTANT

Key:

^a^ Studies point in different directions.

^b^ Some studies are small, and some studies have broad confidence intervals.

Abbreviations: CI: confidence interval; MD: mean difference; SMD: standardized mean difference.

## Discussion

We found that empathy-based teaching interventions improve students’ attitudes towards older persons. Traditional teaching methods alone may increase negative attitudes, and the positive effect observed across all interventions is likely attributed to empathy-based interventions.

The most common empathy-based interventions increase empathy through exposure to relatively healthy and independent older persons, as seen in the studies of Adelman et al., Alford et al., De la Garza et al., Gonzales et al., Lu et al., Shue et al., and Westmoreland et al. Interventions including components designed to increase empathy through sensory deprivation were performed by Intrieri et al. and Warren et al., Eskildsen et al. simulated patient cases, and the computer program of van de Pol et al. involved weighing medical choices against the individual’s preferences. Duke et al. performed interviews with a reflective paper, and Morgan et al. without a reflective paper.

Other empathy-based interventions were knowledge-based and differed in effect. Jeste et al. reported improved attitudes among students participating in mentored research programs. The majority of the research persons were students with positive attitudes pre-intervention, and the program decreased the gap between the attitudes of female and male students. However, the program enrolled students who arguably had a higher baseline interest in geriatrics, which may have affected the results. Hughes et al. assessed traditional teaching methods, daily tutorials, and a project on specific topics without a significant effect on student attitudes. Two programs revealed an increase in negative attitudes, where de Biasio et al. implemented a two-year longitudinal geriatrics clinical experience and Kusumastuti et al. an exposure to older persons with higher degrees of morbidity and functional impairments.

Our study supports previous studies reporting that medical students’ attitudes are related to exposure to relatively healthy and independent older persons in the community as well as through geriatric education [51]. Empathy–building interventions seem to be more effective in improving students’ attitudes [28]. While attitudes are likely to be connected to knowledge levels, and new knowledge in a population has been shown to be one of the most effective methods of changing these attitudes [7], education alone does not seem to be enough.

The funnel plot was not perfectly symmetrical regarding studies with negative results; see Figure 6, suggesting a potential risk of publication bias.

The most commonly used questionnaires were the ASD [29,30,34,43,44,48] and the UCLA-GAS [35,36,39,41,45,50]. The ASD specifically quantifies attitudes and is the most widely used instrument in studies examining medical students’ attitudes [52]. The UCLA-GAS has been criticized for measuring the beliefs rather than attitudes of US medical students; however, it has good construct validity and is widely used internationally [52]. The difference in the pooled meta–analyses that included and excluded the UCLA–GAS may suggest that it is not appropriate to pool other questionnaires with the UCLA–GAS. The validity of the FSA, OP and MSAS have been questioned, as has the COCOA outside of a North Carolina context [52].

Most studies were performed in the United States, where the time assigned to geriatrics is relatively short, which may affect the results [53]. In systematic searches examining determinants of ageism and interventions against ageism, a majority of the studies are from the US [8,54]. Therefore, the results of students’ attitudes may reflect North American perspectives. There are relatively few studies performed in other countries [28]. A previous study explored university students in North America, where half of the students were from other countries [14]. Both groups perceived older adults as individuals who should be respected. North American students viewed older adults as important role models, who need extra help and provide wisdom, love and support. International students viewed them as leaders of the family, whose families are responsible for helping, and that older persons provide knowledge, experience, and opinions that are valued by the family and society. North Americans, however, described negative perceptions and experiences with older adults, while international students did not.

Exposure to geriatric curricula and older persons was performed at various timepoints during education. While exposure later in the medical program seems to be favorable, a longitudinal design shows less improvement in attitudes, possibly representing worsening attitudes during medical education. Samra et al. examined exposure to older persons in a previous systematic review, proposing that the quality of contact or relationships, and not the frequency, is of importance [55].

This study has several limitations. We compared different interventions and instruments in a comprehensive analysis yielding results in a pooled standardized mean difference, which may explain the high heterogeneity measured as I^2^ ranging from 75% to 85%. As this can affect our confidence in the effect estimates, the results should be interpretated with caution. Describing mean differences through ‘rules of thumb’, would mean that the interventions have a small effect. While such descriptions may aid the reader to quantify the effects of interventions, the importance of a finding is context‐dependent [56]. There is also a risk of self-selection bias, as volunteering students may hold more positive attitudes before the intervention. Its strengths are the analysis of a homogenous sample with high response rates. The systematic search was also performed by a trained information specialist, and we included studies from the reference list of previous reviews, reducing the possibility of missing relevant studies to include in the synthesis.

## Conclusion

Empathy-based interventions seem to improve medical students’ attitudes towards older persons. Traditional teaching methods may have no significant effect and may even increase negative attitudes. Although the certainty of the results of this meta-analysis is graded as very low, we suggest that the medical curriculum could benefit from an add-on of empathy-based components to combat ageism. More studies are needed to examine whether the results of studies from North America are valid globally.

## Supplementary Material

Supplementary material1_Search strategy.docx Supplementary material1_Search strategy.docx

Supplementary material3_CASP checklist.docx Supplementary material3_CASP checklist.docx

Supplementary material2_Exluded articles.docx Supplementary material2_Exluded articles.docx

PRISMA_2020_checklist_students_attitudes.docx PRISMA_2020_checklist_students_attitudes.docx

## Data Availability

Data will be made available on reasonable request to the corresponding author.
