# Supplementary material for: Medical students’ attitudes towards older persons – a systematic review and meta-analysis
Source: Med Educ Online. 2026 Apr 21;31(1):2661455. doi: 10.1080/10872981.2026.2661455 (PMC13101001; doi:10.1080/10872981.2026.2661455)
Supplement: Supplementary material1_Search strategy.docx [file ZMEO_A_2661455_SM8172.docx]

Search strategy

| Original search, Medline through OVID 2023-10-12 |  |
| --- | --- |
| Search terms | Results |
| Medical Students |  |
| 1. exp Students, Medical/ OR "medical student*".ab,ti. OR "student physician*".ab,ti. OR "student doctor*".ab,ti. | 69540 |
| Aged |  |
| 1. exp Aged/ OR Aging/ OR "Old* person*".ab,ti. OR "old* patient*".ab,ti. OR "old* adult*".ab,ti. OR "old* people*".ab,ti. OR elder*.ab,ti. OR frail.ab,ti. OR aging.ab,ti. OR ageing.ab,ti. OR "aged patient*".ab,ti. OR "aged person*".ab,ti. OR "geriatric patient*".ab,ti. OR "geriatric person*".ab,ti. OR "old age*".ab,ti. OR "senior citizen*".ab,ti. OR "senior adult*".ab,ti. OR "senior person*".ab,ti. OR "senior patient*".ab,ti. | 3912095 |
| Attitude |  |
| 1. Attitude/ OR "Attitude of Health Personnel"/ OR attitud*.ab,ti. OR belief*.ab,ti. OR Ageism/ OR ageism.ab,ti. OR ageist.ab,ti. OR discriminat*.ab,ti. OR prejudic*.ab,ti. OR stereotyp*.ab,ti. OR stigma.ab,ti. OR label*ing.ab,ti. OR "age bias".ab,ti. | 939390 |
| Combined sets |  |
| 1. 1 AND 2 AND 3 | 655 |
| Limit English |  |
| 1. limit 40 to english language | 624 |

| Updated search, Medline through OVID 2024-08-23 |  |
| --- | --- |
| Search terms | Results |
| Medical Students |  |
| 1. exp Students, Medical/ OR "medical student*".ab,ti. OR "student physician*".ab,ti. OR "student doctor*".ab,ti. | 73796 |
| Aged |  |
| 1. exp Aged/ OR Aging/ OR "Old* person*".ab,ti. OR "old* patient*".ab,ti. OR "old* adult*".ab,ti. OR "old* people*".ab,ti. OR elder*.ab,ti. OR frail.ab,ti. OR aging.ab,ti. OR ageing.ab,ti. OR "aged patient*".ab,ti. OR "aged person*".ab,ti. OR "geriatric patient*".ab,ti. OR "geriatric person*".ab,ti. OR "old age*".ab,ti. OR "senior citizen*".ab,ti. OR "senior adult*".ab,ti. OR "senior person*".ab,ti. OR "senior patient*".ab,ti. | 4026060 |
| Attitude |  |
| 1. Attitude/ OR "Attitude of Health Personnel"/ OR attitud*.ab,ti. OR belief*.ab,ti. OR Ageism/ OR ageism.ab,ti. OR ageist.ab,ti. OR discriminat*.ab,ti. OR prejudic*.ab,ti. OR stereotyp*.ab,ti. OR stigma.ab,ti. OR label*ing.ab,ti. OR "age bias".ab,ti. | 983073 |
| Combined sets |  |
| 1. 1 AND 2 AND 3 | 685 |
| Limit English |  |
| 1. limit 40 to english language | 644 |
| 1. limit 4 to dt="20231012-20240823" | 20 |
| 1. limit 4 to ez="20231012-20240823" | 20 |
| 1. limit 4 to ed="20231012-20240823" | 18 |
| Combined sets |  |
| 1. 6 OR 7 OR 8 | 26 |
