# Supplementary material for: Medical students’ attitudes towards older persons – a systematic review and meta-analysis
Source: Med Educ Online. 2026 Apr 21;31(1):2661455. doi: 10.1080/10872981.2026.2661455 (PMC13101001; doi:10.1080/10872981.2026.2661455)
Supplement: Supplementary material3_CASP checklist.docx [file ZMEO_A_2661455_SM8169.docx]

| Author | Year | Title | Clear focused issue | Appropriate method | Acceptable recruitment | Data collection adresses issue | Enough participants power calc | How results are presented- main results | Analysis rigorous | Clear statement of findings | Results appliable | Valuable research | Appraisal | Unknowns |
| --- | --- | --- | --- | --- | --- | --- | --- | --- | --- | --- | --- | --- | --- | --- |
| Adelman | 1992 | Geriatric education Part II: The effect of a Well Elderly Program on Medical Student Attitudes toward Geriatric Patients | Y | Y | Y (mandatory, students from municipal hospital were excluded as they had access to both sites). | Y | N | Y | Y | Y | Y | Y | Good |  |
| Alford | 2001 | An Introduction to Geriatrics for First-Year Medical Students | Y | Y | Y | Y | Y | Y | Y | Y | Y | Y | Good |  |
| Biasio | 2016 | Longitudinal assessment of medical student  attitudes toward older people | Y | Y | ?  Optional | Y | Y | Y | Y | Y | Y | Y | Good |  |
| Duke | 2009 | Using a Geriatric Mentoring Narrative Program to  Improve Medical Student Attitudes Towards the  Elderly | Y | Y | Y | Y | Y | Y | Y | Y | Y | Y | Good |  |
| De la Garza | 2018 | Evaluation of the Impact of a senior mentor  program on medical students’ geriatric knowledge  and attitudes toward older adults | Y | Y | Y | Y | Y | Y | Y | Y | Y | Y | Good |  |
| Author | Year | Title | Clear focused issue | Appropriate method | Acceptable recruitment | Data collection adresses issue | Enough participants power calc | How results are presented- main results | Analysis rigorous | Clear statement of findings | Results appliable | Valuable research | Appraisal | Unknowns |
| Edwards | 1996 | Attitudes to and knowledge about elderly people: a comparative analysis of students of Medicine, English and Computer Science and their teachers | Y | Y | Y | Y | Y | Y | Y | Y | Y | Y | Good |  |
| Eskildsen | 2009 | A Multimodal Aging and Dying Course for First-Year Medical  Students Improves Knowledge and Attitudes | Y | Y | Y (mandatory) | Y | Y | Y | Y | Y | Y | Y | Good |  |
| Gonzales | 2010 | Changing Medical Students' Attitudes Toward  Older Adults | Y | Y | ? (not mandatory) | Y | Y | Y | Y | Y | Y | Y | Good |  |
| Hughes | 2008 | Medical Student Attitudes Toward Older People and Willingness  to Consider a Career in Geriatric Medicine | Y | Y | Y | Y | Y | Y | Y | Y | Y | Y | Good |  |
| Intrieri | 1993 | Improving Medical Students’ Attitudes Toward and Skills with the Elderly | Y | Y | Y | Y | N | Y | Y | Y | Y | Y | Good |  |
| Author | Year | Title | Clear focused issue | Appropriate method | Acceptable recruitment | Data collection adresses issue | Enough participants power calc | How results are presented- main results | Analysis rigorous | Clear statement of findings | Results appliable | Valuable research | Appraisal | Unknowns |
| Kusumastuti | 2017 | When Contact Is Not Enough: Affecting First  Year Medical Students' Image towards Older  Persons | Y | Y | Y (compulsory) | Y | Y | Y | Y | Y | Y | Y | Good |  |
| Lu | 2010 | First Year Medical Students' Knowledge, Attitudes,  and Interest in Geriatric Medicine | Y | Y | N (voluntary to participate in STEP program) | Y | Y | Y | Y | Y | Y | Y | Good |  |
| Jeste | 2018 | Effect of Short-Term Research Training Programs on Medical Students’ Attitudes Toward Aging | Y | Y | N (voluntary, specific criteria for MSTAR) | Y | Y | Y | Y | Y | Y | Y | Good |  |
| Morgan | 2024 | Combating ageism in medical education with  narrative medicine | Y | Y | Y | Y | Y | Y | Y | Y | Y | Y | Good |  |
| van de Pol | 2014 | Teaching Geriatrics Using an Innovative, Individual-Centered  Educational Game: Students and Educators Win. A Proof-of-  Concept Study | Y | Y | N (voluntary participation, elective course) | Y | N | Y | Y | Y | Y | Y | Good |  |
| Author | Year | Title | Clear focused issue | Appropriate method | Acceptable recruitment | Data collection adresses issue | Enough participants power calc | How results are presented- main results | Analysis rigorous | Clear statement of findings | Results appliable | Valuable research | Appraisal | Unknowns |
| Powell | 1988 | Stability of Medical Students’ Attitudes Toward Aging and Death | Y | ? (intervention was medical school, unadjusted) | Y | Y | Y | Y | Y | Y | Y | Y | Good |  |
| Ruiz | 2015 | Group-Based Differences in Anti-Aging Bias Among  Medical Students | Y | Y | N (e-mail, voluntary) | Y | Y | Y | Y | Y | Y | Y | Good |  |
| Sainsbury | 1992 | Attitudes of medical students to old people: a cross-sectional national comparative study | Y | Y | Y | Y | Y | Y | Y | Y | Y | Y | Good |  |
| Shue | 2005 | Changing Medical Students’ Attitudes about  Older Adults and Future Older Patients | Y | Y | Y | Y | Y | Y | Y | Y | Y | Y | Good |  |
|  |  |  |  |  |  |  |  |  |  |  |  |  |  |  |
| Author | Year | Title | Clear focused issue | Appropriate method | Acceptable recruitment | Data collection adresses issue | Enough participants power calc | How results are presented- main results | Analysis rigorous | Clear statement of findings | Results appliable | Valuable research | Appraisal | Unknowns |
| Warren | 1983 | Effects of Geriatric Education on the Attitudes of Medical Students | Y | Y | Y | Y | Y (n) | Y | Y | Y | Y | Y | Good |  |
| Westmoreland | 2009 | Improving Medical Student Attitudes Toward Older Patients  Through a ‘‘Council of Elders’’ and Reflective Writing Experience | Y | Y | Y | Y | Y | Y | Y | Y | Y | Y | Good |  |
