# Supplementary material for: Medical students’ attitudes towards older persons – a systematic review and meta-analysis
Source: Med Educ Online. 2026 Apr 21;31(1):2661455. doi: 10.1080/10872981.2026.2661455 (PMC13101001; doi:10.1080/10872981.2026.2661455)
Supplement: Supplementary material2_Exluded articles.docx [file ZMEO_A_2661455_SM8170.docx]

Appendix 2. Excluded articles and reasons for exlusion

| Author | Year | Title | Reason for exclusion |
| --- | --- | --- | --- |
| Adelman et al. | 1987 | Medical Student Attitudes Toward the Elderly: A Critical Review of the Literature | Review article |
| Alqahtani et al. | 2022 | A cross-sectional study: exploring  knowledge and attitude of medical and nursing students to Care for Elders in the future | No intervention(s) |
| Atkinson et al. | 2013 | Teaching Medical Student Geriatrics Competencies in 1 Week: An Efficient Model to Teach and Document Selected  Competencies Using Clinical and Community Resources | Statistical dispersion not reported |
| Ayoğlu et al. | 2014 | Attitudes of Turkish Nursing and Medical Students Toward Elderly People | No intervention(s) |
| Béland et al. | 1990 | Medical Care for the Elderly- Attitudes of Medical Caregivers | No intervention(s) |
| Bensadon et al. | 2013 | Attitude Adjustment: Shaping Medical Students’ Perceptions of Older Patients with a Geriatrics Curriculum | Statistical dispersion not reported |
| Bernard et al. | 2003 | An Evaluation of a Low-Intensity Intervention to Introduce Medical Students to Healthy Older People | Statistical dispersion not reported |
| Brooks et al. | 1991 | Attitudes of Medical Students and Family Practice Residents Toward Geriatric Patients | No intervention(s) |
| Cankurtatan et al. | 2006 | Influence of Medical Education on Students' Attitudes towards the Elderly | Statistical dispersion not reported |
| Chróinín et al. | 2013 | Would you be a geriatrician? Student career preferences and attitudes to a career in geriatric  medicine | No measure of attitudes towards older persons |
| Chua et al. | 2008 | Attitudes of First-year Medical Students in Singapore Towards Older People and Willingness to Consider a Career in Geriatric Medicine | No intervention(s) |
| Denton et al. | 2009 | A Prospective Controlled Trial of the Influence of a Geriatrics Home  Visit Program on Medical Student Knowledge, Skills, and Attitudes  Towards Care of the Elderly | No measure of attitudes before and after intervention |
| Dorfman et al. | 2008 | Incorporating Intergenerational Service-Learning into an Introductory Gerontology Course | Sample does not include medical students |
| Draper et al. | 2024 | How do ageism, death anxiety and ageing anxiety among medical students and residents  affect their attitude towards medical care for older patients: a systematic review | Review article |
| Fitzgerald et al. | 2003 | Relating Medical Students’ Knowledge, Attitudes, and experience to an Interest in Geriatric Medicine | No intervention(s) |
| Galinsky et al. | 1985 | Ten Years’ Experience Teaching Geriatric Medicine | No quantitative measures |
| Ghailani, Al et al. | 2024 | Knowledge and Attitude Towards the Elderly Among Doctors and Medical Students: A Questionnaire-Based Study | No intervention(s) |
| Geiger et al. | 1978 | Note: How Future Professionals View the Elderly: A Comparative Analysis of Social Work, Law, and Medical Students’ Perceptions | No intervention(s) |
| Geijer et al. | 2022 | Walking a mile in Grandma’s shoes – medical students’ evaluation of a very simple online  aging game to enhance their understanding of older patients | No quantitative measures |
| Goeldlin et al. | 2014 | Effects of geriatric clinical skills training on the  attitudes of medical students | Results not reported in a suitable form for meta-analysis |
| Golden et al. | 2010 | A Fourth-Year Medical School Clerkship That Addressed  Negative Attitudes Toward Geriatric Medicine | No measure of attitudes towards older persons |
| Golden et al. | 2013 | The attitudes of graduate healthcare students toward older adults, personal aging, health care  reform, and interprofessional collaboration | No intervention(s) |
| Green et al. | 1983 | Medical Students’ Attitudes Toward the Elderly | Statistical dispersion not reported |
| Hall et al. | 1997 | Longitudinal Effects of a Medical School Geriatrics Clerkship | No measure of attitudes towards older persons |
| Halpert et al. | 1981 | The Student Perspective on the Teaching of Geriatrics in Medical School | No quantitative measures |
| Higashi et al. | 2012 | Elder care as “frustrating” and “boring”: Understanding the persistence of negative attitudes toward older patients among physicians-in-training | No quantitative measures |
| Holtzman et al. | 1979 | Specialty Preference and Attitudes Toward the Aged | No intervention(s) |
| Hylton et al. | 2003 | Knowledge of Aging and Attitudes Toward Older People- A Survey of Australian Podiatric Medical Students | No intervention(s) |
| Jester et al. | 2019 | Beliefs Regarding Geriatrics Primary Care Topics Among Medical Students and  Internal Medicine Residents | Abstract only |
| Jester et al. | 2020 | Attitudes toward aging of health professions  students: Implications for geriatrics education | No intervention(s) |
| Karikari et al. | 2020 | Stimulators of medical students’ interest in geriatric medicine—A  Systematic Review | Review article |
| Lee et al. | 2019 | Ageism between Medical and Preliminary Medical Persons in Korea | No intervention(s) |
| Li Chu et al. | 2020 | Attitudes Toward Aging: A Glance Back at Research Developments Over the Past 75 Years | Review article |
| Linn et al. | 1987 | Predicting Third Year Medical Students’ Attitudes Toward the Elderly and Treating the Old | No intervention(s) |
| Mamcarz et al. | 2020 | The role of emotional intelligence in attitudes towards elderly patients – Comparative study of  medical students from rural and urban areas | No intervention(s) |
| McCarthy et al. | 2021 | An exploration of medical student attitudes towards older persons  and frailty during undergraduate training | No intervention(s) |
| Meiboom et al. | 2015 | Why medical students do not choose a career in geriatrics: a systematic review | Review article |
| Merrill et al. | 1995 | Measuring Social Desirability Among Senior Medical Students | No measure of attitudes towards older persons |
| Merrill et al. | 1996 | Self-esteem and Caregivers’ Attitudes Toward Elderly Persons | No intervention(s) |
| Merrill et al. | 1998 | Medical Manners: Medical Students’ Perceptions of Their Own | No measure of attitudes towards older persons |
| Miller et al. | 1990 | Medical Student Attitudes Toward Elderly Patients: Effects of Social Attractiveness | Aims and methods differ from inclusion criteria |
| Milutinović et al. | 2015 | Knowledge and attitudes of health care science students toward older people | No intervention(s) |
| Muangpaisan et al. | 2008 | Attitudes of Medical Students and Residents Toward Care of the Elderly | No intervention(s) |
| Muntsant et al. | 2021 | The Spanish Intergenerational Study: Beliefs, Stereotypes,  and Metacognition about Older People and Grandparents to  Tackle Ageism | No quantitative measures |
| Ogenler et al. | 2012 | The Views of Medical Students on Elder Discrimination | No intervention(s) |
| Peach et al. | 1982 | Attitudes towards the care of the aged and to a career with elderly patients among students attached to a geriatric and general medical firm | Statistical dispersion not reported |
| Perotta et al. | 1981 | Medical Student Attitudes Toward Geriatric Medicine and Patients | No intervention(s) |
| Powell et al. | 1983 | Attitudes of Medical Students | Letter to the editor |
| Rashti et al. | 1994 | Ageism Distorts How Medical Students (and Doctors?) See and Assess Patients | No quantitative measures |
| Reube et al.n | 1995 | Attitudes of Beginning Medical Students Toward Older Persons: A Five-Campus Study | No intervention(s) |
| Roberts et al. | 2006 | The Senior Methods Program at the University of South Carolina School of Medicine: An Innovative Geriatric Longitudinal Curriculum | Results of measurements of attitudes not reported |
| Ross et al. | 2017 | Improving health care student attitudes toward older adults through educational interventions: A systematic review | Review article |
| Rull et al. | 2009 | Aging Couple Across the Curriculum | Results not reported in a suitable form for meta-analysis |
| Sainsbury et al. | 1994 | Do clinical years change medical students’ attitudes to old people? | Sample does not include medical students |
| Samra et al. | 2013 | Changes in Medical Student and Doctor Attitudes Toward  Older Adults After an intervention: A Systematic Review | Review article |
| Samra et al. | 2015 | Medical students’ and doctors’ attitudes towards older patients and their care in hospital settings: a conceptualisation | No quantitative measures |
| Samra et al. | 2017 | Factors related to medical students’ and doctors’ attitudes towards older patients: a systematic review | Review article |
| Santos, Dos et al. | 2011 | Perception of Health Students About Older Adults in Brazil | No intervention(s) |
| Schüttengruber et al. | 2021 | Attitudes towards older adults (80 years and older): A measurement with the ageing semantic differential -A  cross-sectional study of Austrian students | No intervention(s) |
| Schigelone et al. | 2010 | Some of my Best Friends are Old: a Qualitative Exploration of Medical Students’ Interest in Geriatrics | No quantitative measures |
| Sema et al. | 2024 | Knowledge and Attitudes of Graduating Medical, Pharmacy, and Nursing Students Toward Geriatric Care at the College of Medicine and Health Sciences,  University of Gondar, North West Ethiopia | No intervention(s) |
| Shahidi et al. | 1993 | Medical students’ attitudes to and knowledge of the aged | No intervention(s) |
| Smith et al. | 1989 | Medical students’ attitudes to old people and career preference: the case of Nottingham Medical School | Statistical dispersion not reported |
| Snyder et al. | 2010 | Medical and Psychology Students’ Knowledge and Attitudes Regarding Aging and Sexuality | No intervention(s) |
| Solomon et al. | 1979 | Attitudes of Health Workers Toward Old People | Results not divided into subgroups of students |
| Somers et al. | 1978 | Teaching Geriatric Care: Report on an Experimental  Second-Year Elective | No quantitative measures |
| Spence et al. | 1968 | Medical Student Attitudes Toward the Geriatric Patient | No intervention(s) |
| Tandon et al. | 2010 | Changing medical students’ attitudes about ageing  and health | Abstract only |
| Tarbox et al. | 1987 | Freshman and Senior Medical Students’ Attitudes Toward the Elderly | Statistical dispersion not reported |
| Ten Haken et al. | 1995 | A Longitudinal Investigation of Changes in Medical Students’ Attitudes Toward the Elderly | Statistical dispersion not reported |
| Thorson et al. | 1991 | Medical student’s attitudes towards ageing and death: a cross-sectional study | Duplicate of results |
| Tullo et al. | 2010 | Systematic Review: Helping the Young to Understand the Old.  Teaching Interventions in Geriatrics to Improve the Knowledge, Skills, and Attitudes of Undergraduate Medical Students | Review article |
| Ubachs-Moust et al. | 2008 | Value judgements in the decision-making process for  the elderly patient | No measure of attitudes towards older persons |
| Wang et al. | 2009 | Taiwanese Medical and Nursing Student Interest Levels in and Attitudes Towards Geriatrics | No intervention(s) |
| Wattis et al. | 1986 | Medical students’ attitudes to old people and career preference: a comparison of two universities | No intervention(s) |
| Wilkinson et al. | 2002 | The earlier, the better: the effect of early community contact  on the attitudes of medical students to older people | Statistical dispersion not reported |
| Wilson et al. | 2018 | Medical student attitudes towards older  people: a critical review of quantitative  measures | Review article |
| Wilson et al. | 2018 | Understanding Australian medical student attitudes towards older people | Review article |
| Wong et al. | 2009 | First-year Medical Students’ Attitudes Towards the Elderly in Singapore and Clinical Specialty Preferences | Letter to the editor |
| Zambrini et al. | 2008 | Attitudes Toward the Elderly Among Students of Health Care Related Studies at the University of Salamanca, Spain | No intervention(s) |
| Zuilen, Van et al. | 2001 | Medical Students' Positive and Negative  Misconceptions About the Elderly | No measure of attitudes towards older persons |
